# Efficacy and safety of total glucosides of paeony as an add-on treatment in adolescents and adults with chronic urticaria: A systematic review and meta-analysis

**DOI:** 10.3389/fphar.2022.961371

**Published:** 2022-09-23

**Authors:** Ming Li, Yan Li, Lujing Xiang, Linfeng Li

**Affiliations:** Department of Dermatology, Beijing Friendship Hospital, Capital Medical University, Beijing, China

**Keywords:** chronic urticaria, total glucosides of paeony, efficacy, safety, meta-analysis, randomized controlled trial

## Abstract

**Background:** Total glycosides of paeony (TGP), an active compound extracted from the dried roots of *Paenoia lactiflora* Pall., has been widely used to treat chronic urticaria (CU) in China. This study aims to systematically evaluate the efficacy and safety of TGP as an add-on treatment for the treatment of CU in adolescents and adults.

**Methods:** Eight literature databases and two clinical trial registries were searched from their inception to 31 May 2022. Randomized controlled trials on TGP as an add-on treatment for CU in adolescents and adults were included. The Cochrane Collaboration’s risk of bias tool was used for the methodological quality assessment, and RevMan 5.3 software and Stata 12.0 software were used for data analyses.

**Results:** A total of 30 studies with 2,973 participants were included in this meta-analysis. The methodological qualities of all included studies were suboptimal. The pooled results showed that TGP combined with H1-antihistamine was superior to H1-antihistamine alone in the cure rate (risk ratio (RR) = 1.54, 95% confidence interval (CI) = 1.39 to 1.71, *p* < 0.00001), total efficacy rate (RR = 1.33, 95%CI = 1.26 to 1.40, *p* < 0.00001), urticaria activity score 7 (mean difference (MD) = -4.03, 95%CI = -6.62 to -1.44, *p* = 0.002), recurrence rate (RR = 0.31, 95%CI = 0.20 to 0.46, *p* < 0.00001), and the level of IgE in serum (standardized mean difference (SMD) = -1.96, 95%CI = -3.02 to -0.90, *p* = 0.0003). In terms of safety, the incidence of diarrhea (RR = 6.19, 95%CI = 3.39 to 11.29, *p* < 0.00001) was significantly increased in the TGP plus H1-antihistamine groups, and no abnormal results of laboratory tests and electrocardiogram were reported in two groups. The qualities of evidences were evaluated as moderate to low.

**Conclusions:** TGP as an add-on treatment could provide a good effect for CU in adolescents and adults with mild and tolerable adverse events. However, in view of poor methodological quality, high-quality and long-term clinical trials are needed in the future to confirm and update the evidence.

## 1 Introduction

Chronic urticaria (CU) is a common skin disease characterized by the recurrent appearance of wheals, angioedema, or both for more than 6 weeks. There are considerable regional differences in the prevalence of CU, and the overall lifetime prevalence reaches up to 1.4% (Fricke et al., 2020). Because of severe itching and recurrent lesions, CU causes some significant impacts on patients’ quality of life, including sleep disturbance and social dysfunction. Moreover, the economic burdens for patients and healthcare systems are also substantial (Itakura et al., 2018; Williams et al., 2018). In the guidelines for the treatment of CU, H1-antihistamine is recommended as the first-line treatment. However, some patients are still not responsive to H1-antihistamine even at fourfold standard dose. When H1-antihistamine fails to achieve an adequate response, some different agents, including omalizumab, cyclosporine, and systemic corticosteroids, could be added (He et al., 2021). However, the high cost and adverse effects, such as infection, elevated blood pressure, and renal dysfunction, restrict the clinical application of these agents in patients with CU (Matsubara et al., 2021).

In China, total glucosides of paeony (TGP) is an active compound extracted from dried roots of *Paenoia lactiflora* Pall. (Baishao in Chinese). Some pharmacological studies have demonstrated the anti-inflammatory and immunomodulatory effects of TGP (Jiang et al., 2020). In clinical practice, TGP has been widely applied for some autoimmune and inflammatory diseases, such as rheumatoid arthritis, diabetic kidney disease, Sjögren’s syndrome, psoriasis, and alopecia areata (Yang et al., 2012; Zhu et al., 2016a; Zhou et al., 2016; Zheng et al., 2019; Liu et al., 2021). Despite without the indication of CU, TGP has been used for patients with CU in China in the past decade. Some clinical trials have revealed that TGP combined with H1-antihistamine could significantly improve the efficacy in comparison with H1-antihistamine alone, and no serious adverse reactions were reported, indicating that TGP might be a potential therapeutic agent for CU (Liu 2010; Yuan et al., 2014). In addition, the cost of TGP is much lower than those of omalizumab and cyclosporine.

To our knowledge, one meta-analysis regarding the effectiveness and safety of TGP combined with H1-antihistamine for the treatment of CU has been published (Zhu et al., 2016b). However, this meta-analysis only consisted of the articles until September 2015, and one duplicate study was included. Moreover, the definitions of outcomes were not interpreted, and some important outcomes were not assessed, such as recurrence rate and laboratory tests. Therefore, in order to provide thorough evidence for clinical practice, this meta-analysis planned to re-evaluate and update the efficacy and safety of TGP as an add-on treatment for adolescent and adult patients with CU with regard to the latest published articles.

## 2 Methods

This systematic review and meta-analysis was conducted in accordance with the Preferred Reporting Items for Systematic reviews and Meta-Analyses (PRISMA) guidelines (Liberati et al., 2009), and the protocol was registered at PROSPERO (Registration number: CRD42021243815).

### 2.1 Search strategy

Four Chinese literature databases and four English literature databases were searched from their inception to 31 May 2022, including China National Knowledge Infrastructure (CNKI), Wanfang Database (Wanfang), Chinese Scientific Journal Database (VIP), Chinese Biomedical Database (SinoMed), PubMed, Cochrane library, Embase and Web of Science. In addition, the following two clinical trial registries were also searched: ClinicalTrials. gov and Chinese Clinical Trial Registry.

The following search strategy was set based on the PubMed database, which was applicable to other databases as well: (urticaria [MeSH Terms] OR urticaria [Abstract/Title]) AND (total glucosides of paeony [Abstract/Title] OR TGP [Abstract/Title] OR Pafulin [Abstract/Title] OR Pa Fu Lin [Abstract/Title] OR Baishao Zonggan [Abstract/Title] OR Bai Shao Zong Gan [Abstract/Title] OR Baishaozonggan [Abstract/Title] OR Baishao Zongdai [Abstract/Title] OR Bai Shao Zong Dai [Abstract/Title] OR Baishaozongdai [Abstract/Title]).

### 2.2 Inclusion criteria

#### 2.2.1 Types of studies

Only randomized controlled trials (RCTs) were included.

#### 2.2.2 Types of participants

Based on the medical history and clinical feature, adolescents (aged 12–17 years) and adults (aged >18 years) were diagnosed with CU characterized by presence of wheals and/or angioedema that appear continuously for more than 6 weeks. There were no restrictions on gender, disease course, and disease severity.

#### 2.2.3 Types of interventions and comparisons

The test groups were treated with TGP plus H1-antihistamine, while the control groups were treated with H1-antihistamine alone. There were no restrictions on dose of each drug, frequency of drug administration, and treatment duration.

#### 2.2.4 Types of outcomes

The primary outcomes included cure rate and total efficacy rate. Cure rate was defined as the proportion of patients achieving at least 90% improvement in disease severity from baseline, and total efficacy rate was defined as the percentage of patients achieving ≥60% improvement in disease severity from baseline.

The secondary outcomes included urticaria activity score 7 (UAS7), recurrence rate, the level of immunoglobulin E (IgE) in serum, and adverse events (AEs), including diarrhea, drowsiness, dry mouth, laboratory tests, and electrocardiogram (ECG).

### 2.3 Exclusion criteria

The exclusion criteria were as follows: 1) reviews, case reports, abstracts, animal experiments, cell experiments, and non-RCTs; 2) studies with data errors, studies without detailed data, and duplicate publications; 3) studies published in neither Chinese nor English.

### 2.4 Study selection

Two reviewers (ML and YL) independently conducted literature selection according to the inclusion and exclusion criteria. The retrieved studies were first screened based on the titles and abstracts, and the full texts of potentially eligible studies were obtained to identify the final included studies. If any disagreement was encountered, the third reviewer (LX) was consulted to make the final decision.

### 2.5 Data extraction

The data from each trial were extracted by two independent reviewers (ML and YL), including the first author, publication year, country, the number of research center, methodological information, sample size, age, gender, disease duration, dosage of each drug, treatment duration, and outcome measures. Discrepancies were resolved by consulting with the third reviewer (LX).

### 2.6 Quality assessment

The Cochrane Collaboration’s risk of bias tool (Higgins et al., 2011) was used to evaluate the methodological quality of each included trial. The tool included the following seven domains: random sequence generation (selection bias), allocation concealment (selection bias), blinding of participants and personnel (performance bias), blinding of outcome assessment (detection bias), incomplete outcome data (attrition bias), selective reporting (reporting bias), and other bias. In this meta-analysis, the baselines of disease severity between two groups were considered as the source of other bias. The risk of bias of each domain was judged as “low risk”, “unclear risk” or “high risk.”

The quality of evidence was assessed by using the Grading of Recommendations Assessment, Development and Evaluation (GRADE) approach (Guyatt et al., 2008). The levels of quality were classified into the following four grades: “high”, “moderate,” “low,” or “very low.” The quality rating would fall from high quality to moderate, low or very low quality, depending on the presence of the five factors, including limitations in the design and implementation, inconsistency of results, indirectness of evidence, imprecision of results, and probability of publication bias.

Two reviewers (ML and YL) independently conducted both methodological quality assessment and GRADE assessment. In case of any disagreement, the third author (LX) was consulted.

### 2.7 Statistical analysis

Review Manager 5.3 (RevMan 5.3) software and Stata 12.0 software were utilized for data analyses. Dichotomous data were expressed as risk ratio (RR) with 95% confidence interval (CI), whereas continuous data were expressed as mean difference (MD) or standardized mean difference (SMD) with 95%CI. The I-squared and Chi-squared tests were used to assess statistical heterogeneity. When a significant heterogeneity was identified (*I* ^2^ > 50% or *p* < 0.10), a random-effect model was applied, otherwise a fixed-effect model was used. The subgroup analyses were conducted based on the treatment duration or follow-up time. Moreover, in order to identify the robustness of the pooled result, the sensitivity analysis was performed by omitting each trial at a time or modifying the effect measure to odds ratio (OR). If 10 studies or more were included in the same outcome, the potential publication bias was assessed by using funnel plot, Egger’s test and Begg’s test. All tests were two-sided, and *p* < 0.05 was considered statistically significant.

## 3 Results

### 3.1 Study selection

**FIGURE 1:**
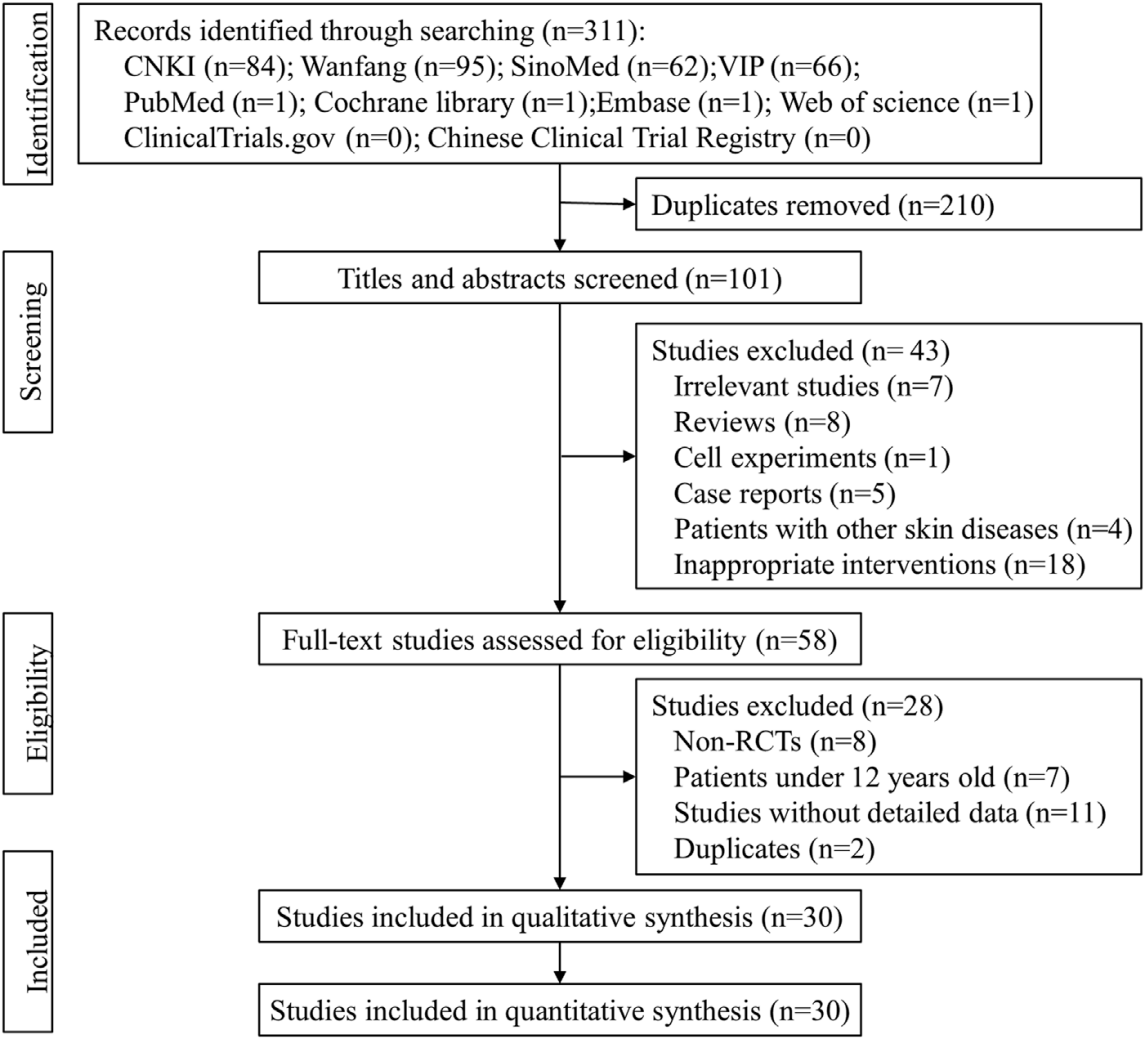
The flow diagram of the study selection process.

According to the search strategy, 311 records were identified from eight literature databases and two clinical trial registries, and 210 repetitive records were excluded. A total of 43 studies were excluded after reading the titles and abstracts, and the full texts of the remaining 58 studies were retrieved for further evaluation. Among them, 28 studies did not meet the eligible criteria and were excluded. Finally, 30 eligible studies (Shi, 2008; Li et al., 2010; Long et al., 2010; Wang and Li, 2011; Guo and Yuan, 2012; Jia et al., 2012; Lin, 2012; Mao, 2012; Ren et al., 2012; Yang, 2012; Liang et al., 2013; Zhang and Zhang 2013; Zou et al., 2013; Zhang et al., 2014a; Zhang et al., 2014b; Sun et al., 2014; Xie, 2014; Yang, 2014; Zhao et al., 2014; Chu, 2015; Liao, 2015; Dong, 2016; Zhang, 2017; Zhu, 2017; Guo, 2018; Lin, 2018; Peng, 2019; Cen, 2020; Zha and Deji, 2020; Deng et al., 2021) were included in this meta-analysis. The literature selection process is shown in Figure 1.

### 3.2 Characteristics of the included studies

All included studies were single-center trials and conducted in China. Except for one study (Long et al., 2010) published in English in 2010, the rest 29 studies were published in Chinese from 2008 to 2021. In this meta-analysis, a total of 2,973 patients aged 12–83 years were included, of which 1,529 patients receiving TGP plus H1-antihistamine and 1,444 patients receiving H1-antihistamine alone. In terms of the dosage of TGP, patients were treated with 600 mg twice per day in nine studies (Mao, 2012; Yang, 2012; Zhang et al., 2014b; Xie, 2014; Yang, 2014; Dong, 2016; Zhu, 2017; Peng, 2019; Cen, 2020), while patients received 600 mg 3 times per day in the rest 21 studies. The treatment durations ranged from 4 to 12 weeks. The most frequently reported outcome was AEs, followed by cure rate, total efficacy rate, recurrence rate, the level of IgE in serum, and UAS7. The characteristics of all included studies are presented in Table 1.

**TABLE 1:** Characteristics of included studies.

Study ID	Sample size (male/female)		Age (years)		Duration of disease (months)		Interventions		Treatment duration (weeks)	Outcomes
	T	C	T	C	T	C	T	C		
Deng et al. (2021)	40 (17/23)	40 (19/21)	19–64	18–65	3–34	2–38	TGP (600 mg tid) + Lupatadine (10 mg qd)	Lupatadine (10 mg qd)	8	①②⑥
Zha and Deji (2020)	36 (20/16)	36 (19/17)	18–63	20–65	NA	NA	TGP (600 mg tid) + Levocetirizine (5 mg qd)	Levocetirizine (5 mg qd)	4	①②⑥
Cen (2020)	100 (42/58)	100 (43/57)	18–54	19–55	6–72	7–72	TGP (600 mg bid) + Desloratadine citrate disodium (8.8 mg qd)	Desloratadine citrate disodium (8.8 mg qd)	4	⑥
Peng (2019)	69 (42/27)	68 (39/29)	17–68	18–69	NA	NA	TGP (600 mg bid) + Levocetirizine (5 mg qd) + Desloratadine (5 mg qd)	Levocetirizine (5 mg qd) + Desloratadine (5 mg qd)	4	⑥
Lin (2018)	30	30	12–70	12–70	NA	NA	TGP (600 mg tid) + Levocetirizine (10 mg qd)	Levocetirizine (10 mg qd)	8	①②⑥
Guo (2018)	45 (22/23)	45 (21/24)	18–65	18–70	2–360	2–336	TGP (600 mg tid) + Levocetirizine (5 mg qd)	Levocetirizine (5 mg qd)	12	①②⑥
Zhu (2017)	65 (40/25)	65 (38/27)	22–63	23–64	6–22	6–23	TGP (600 mg bid) + Desloratadine (5 mg qd) + Levocetirizine (5 mg qd)	Desloratadine (5 mg qd) + Levocetirizine (5 mg qd)	8	①②⑥
Zhang (2017)	35 (11/24)	35 (13/22)	60–83	60–81	10–108	9–132	TGP (600 mg tid) + Mizolastine (10 mg qd)	Mizolastine (10 mg qd)	8	②
Dong (2016)	55 (20/35)	55 (18/37)	19–60	20–60	6–60	5–60	TGP (600 mg bid) + Desloratadine citrate disodium (8.8 mg qd)	Desloratadine citrate disodium (8.8 mg qd)	4	①②⑥
Chu (2015)	80 (38/42)	82 (43/39)	18–60	19–58	2–24	2–30	TGP (600 mg tid) + Acrivastine (8 mg tid)	Acrivastine (8 mg tid)	12	①②⑥
Liao (2015)	41 (13/28)	40 (16/24)	19–58	20–55	NA	NA	TGP (600 mg tid) + Desloratadine citrate disodium (8.8 mg qd)	Desloratadine citrate disodium (8.8 mg qd)	12	①②④⑥
Zhang et al. (2014a)	30 (14/16)	30 (13/17)	13–65	15–62	2–216	3–180	TGP (600 mg tid) + Ebastine (10 mg/qd)	Ebastine (10 mg qd)	8	①②④⑥
Zhao et al. (2014)	44 (20/24)	47 (21/26)	18–60	19–58	2–24	2–36	TGP (600 mg tid) + Fexofenadine (120 mg qd)	Fexofenadine (120 mg qd)	4	①②⑥
Yang (2014)	65 (27/38)	65 (25/40)	18–60	18–55	1.5–12	1.5–12	TGP (600 mg bid) + Desloratadine (5 mg qd)	Desloratadine (5 mg qd)	12	①②⑥
Sun et al. (2014)	88 (36/52)	80 (31/49)	18–66	19–68	2–144	2–120	TGP (600 mg tid) + Fexofenadine (60 mg bid)	Fexofenadine (60 mg bid)	8	①②⑥
Zhang et al. (2014b)	42 (22/20)	42 (23/19)	16–57	17–57.5	3–120	2–108	TGP (600 mg bid) + Mizolastine (10 mg qd)	Mizolastine (10 mg qd)	4	①②⑤
Xie (2014)	100 (51/49)	50 (24/26)	18–50	18–53	5–36	4–36	TGP (600 mg bid) + Desloratadine citrate disodium (8.8 mg qd)	Desloratadine citrate disodium (8.8 mg qd)	4	⑥
Liang et al. (2013)	30 (12/18)	30 (14/16)	18–52	19–55	2–36	3–29	TGP (600 mg tid) + Ebastine (10 mg qd)	Ebastine (10 mg qd)	12	③⑥
Zou et al. (2013)	20	20	18–60	18–60	2–48	2–48	TGP (600 mg tid) + Epinastine (10 mg qd)	Epinastine (10 mg qd)	12	①②⑥
Zhang and Zhang (2013)	45	45	NA	NA	NA	NA	TGP (600 mg tid) + Mizolastine (10 mg qd)	Mizolastine (10 mg qd)	12	⑥
Lin (2012)	26 (9/17)	21 (7/14)	12–59	14–56	2–168	3–132	TGP (600 mg tid) + Cetirizine (10 mg qd)	Cetirizine (10 mg qd)	12	⑥
Mao (2012)	40 (19/21)	40 (22/18)	18–60	18–60	3–60	3–60	TGP (600 mg bid) + Fexofenadine (60 mg bid)	Fexofenadine (60 mg bid)	4	①②④⑥
Guo and Yuan (2012)	62 (28/34)	58 (26/32)	18–65	16–64	2–48	2–36	TGP (600 mg tid) + Ebastine (10 mg qd)	Ebastine (10 mg qd)	4	①②⑥
Ren et al. (2012)	45 (22/23)	45 (20/25)	18–68	18–70	2–384	2–336	TGP (600 mg tid) + Cetirizine (10 mg qd)	Cetirizine (10 mg qd)	12	①②⑥
Yang (2012)	30	30	29–62	29–62	3–192	3–192	TGP (600 mg bid) + Mizolastine (10 mg qd)	Mizolastine (10 mg qd)	4	①②⑥
Jia et al. (2012)	51 (19/32)	51 (22/29)	17–64	19–68	NA	NA	TGP (600 mg tid) + Levocetirizine (5 mg qd)	Levocetirizine (5 mg qd)	8	⑥
Wang and Li (2011)	60 (27/33)	56 (25/31)	18–61	18–59	2–120	3–108	TGP (600 mg tid) + Fexofenadine (60 mg bid)	Fexofenadine (60 mg bid)	4	①④⑤
Li et al. (2010)	30 (13/17)	27 (11/16)	18–62	18–57	3–108	2–84	TGP (600 mg tid) + Mizolastine (10 mg qd)	Mizolastine (10 mg qd)	4	④
Long et al. (2010)	65 (37/28)	55 (32/23)	16–58	18–55	2–60	2–36	TGP (600 mg tid) + Cetirizine (10 mg qd)	Cetirizine (10 mg qd)	4	①②④⑤⑥
Shi (2008)	60 (30/30)	56 (27/29)	16–65	16–65	6–72	6–72	TGP (600 mg tid) + Ebastine (10 mg qd)	Ebastine (10 mg qd)	4	⑥

T, test group; C, control group; TGP, total glucosides of paeony; NA, not available; qd, once per day; bid, twice per day; tid, three times per day.

① Cure rate; ② Total efficacy rate; ③ UAS7; ④ Recurrence rate; ⑤ The level of IgE in serum; ⑥ Adverse events.

### 3.3 Risk of bias of the included studies

**FIGURE 2:**
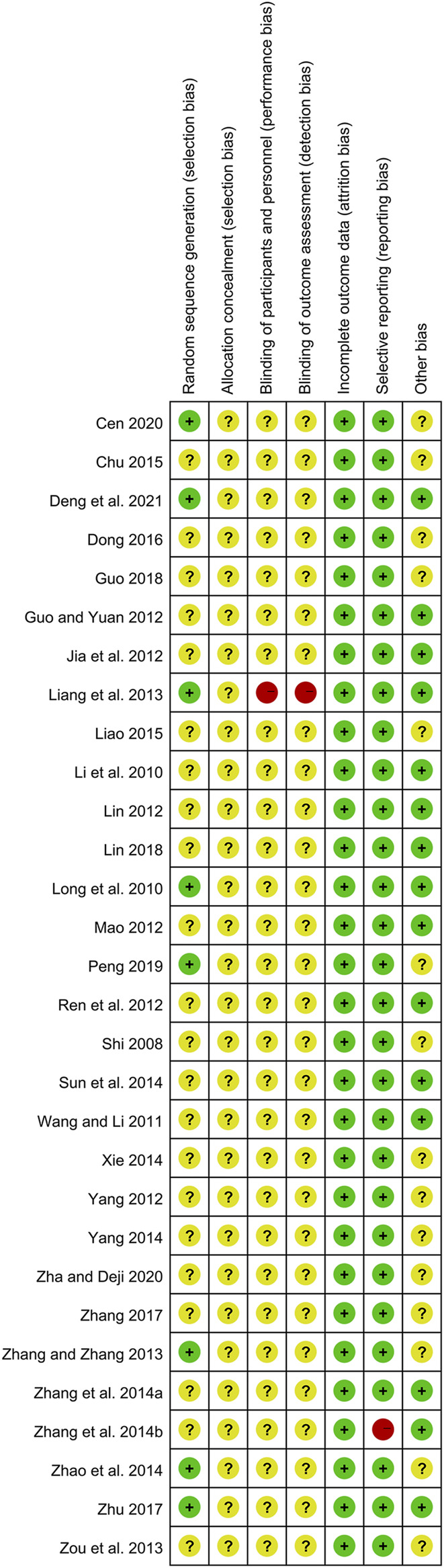
Risk of bias summary of included studies.

**FIGURE 3:**
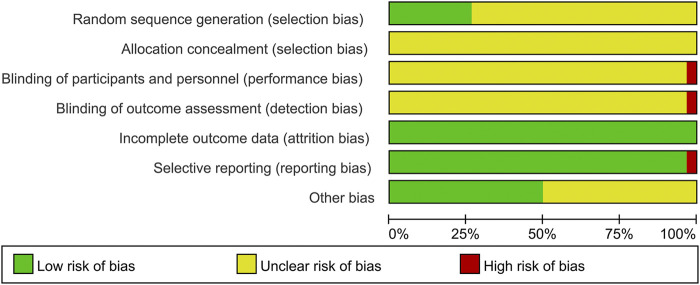
Risk of bias graph of included studies.

Although all included studies declared the randomization, only eight studies reported their methods of generating random sequence and were rated as low risk, including six studies with random number table (Liang et al., 2013; Zhang and Zhang, 2013; Zhao et al., 2014; Zhu, 2017; Cen, 2020; Deng et al., 2021) and two studies with drawing of lots (Long et al., 2010; Peng, 2019). Meanwhile, the rest 22 studies did not describe the detailed methods and were evaluated as unclear risk. Due to the lack of sufficient information, the biases of allocation concealment in all studies were judged as unclear risk. In terms of performance bias and detection bias, except for one open-label trial (Liang et al., 2013) marked as high risk, the remaining 29 studies were judged as unclear risk because they did not mention the blinding. All data of outcomes were available in all studies, and the risks of attribution bias of them were low. With regard to selective reporting, one trial (Zhang et al., 2014b) did not report the results of laboratory tests and had a high risk of bias, while the rest 29 studies were judged as low risk. In the field of other bias, 15 studies (Li et al., 2010; Long et al., 2010; Wang and Li, 2011; Guo and Yuan, 2012; Jia et al., 2012; Lin, 2012; Mao, 2012; Ren et al., 2012; Liang et al., 2013; Zhang et al., 2014a; Zhang et al., 2014b; Sun et al., 2014; Zhu, 2017; Lin, 2018; Deng et al., 2021) provided the data on disease severity between two groups, and they were estimated as low risk. While the remaining 15 studies were judged as unclear risk because they did not show the relevant data. The methodological quality of each trial is shown in Figure 2 and Figure 3.

### 3.4 Primary outcomes

#### 3.4.1 Cure rate

**FIGURE 4:**
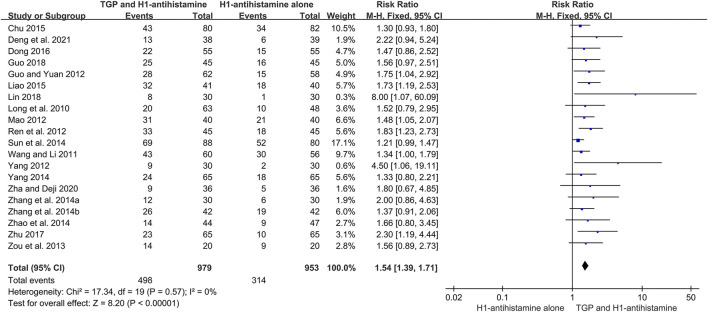
Forest plot of cure rates between TGP combined with H1-antihistamine and H1-antihistamine alone.

A total of 20 studies (Long et al., 2010; Wang and Li, 2011; Guo and Yuan, 2012; Mao, 2012; Ren et al., 2012; Yang, 2012; Zou et al., 2013; Zhang et al., 2014a; Zhang et al., 2014b; Sun et al., 2014; Yang, 2014; Zhao et al., 2014; Chu, 2015; Liao, 2015; Dong, 2016; Zhu, 2017; Guo, 2018; Lin, 2018; Zha and Deji, 2020; Deng et al., 2021) reported the cure rate after 4–12 weeks of treatment, and 1932 patients were enrolled. A fixed-effect model was used due to no significant heterogeneity (*I* ^2^ = 0%, *p* = 0.57). The pooled results showed that TGP combined with H1-antihistamine could significantly improve the cure rate in comparison with H1-antihistamine alone (RR = 1.54, 95%CI = 1.39 to 1.71, *p* < 0.00001) (Figure 4).

**FIGURE 5:**
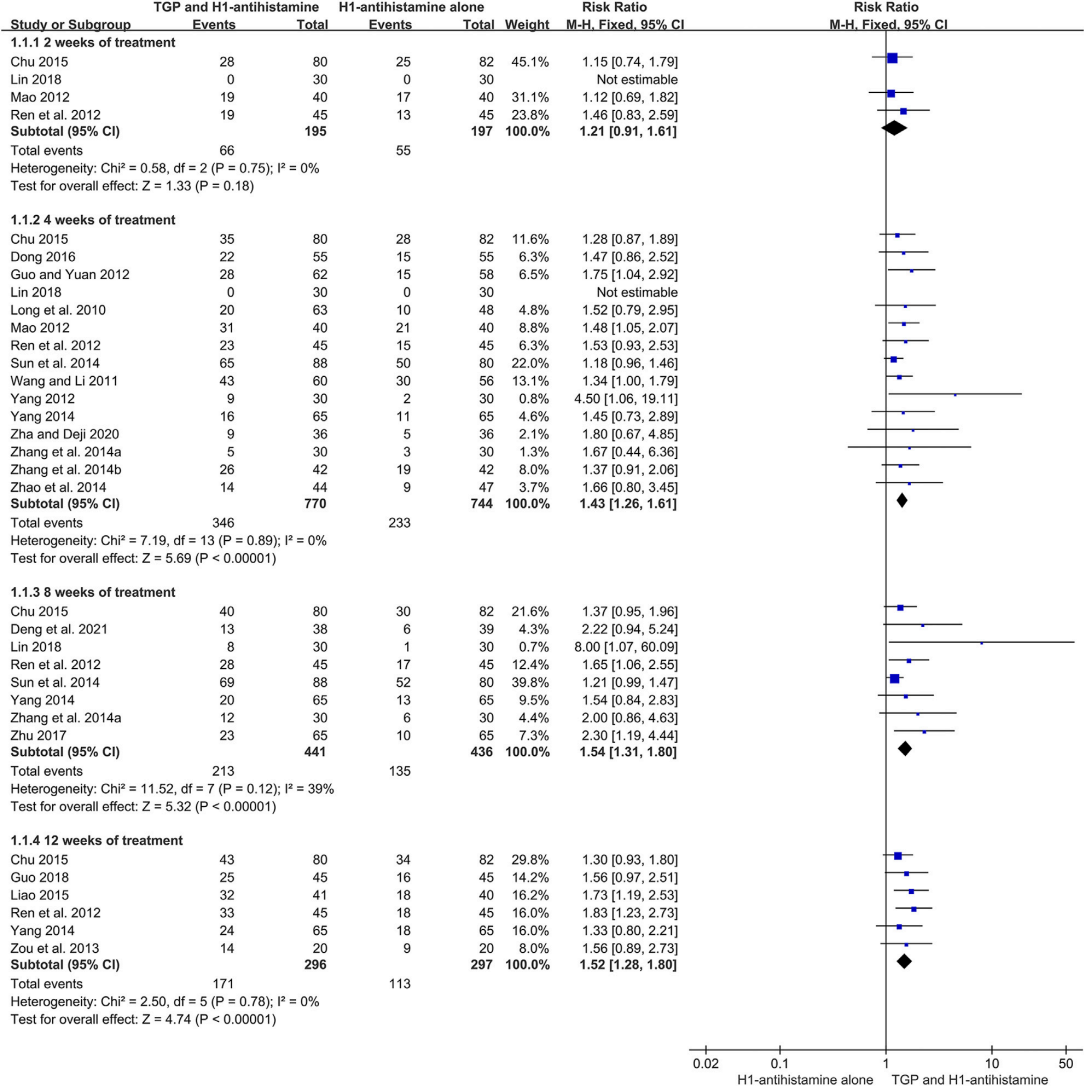
Forest plots of cure rates at week 2, week 4, week 8, and week 12.

According to different treatment durations, a subgroup analysis was conducted. The results showed that there was no significant difference on the cure rates between two groups at week 2 (RR = 1.21, 95%CI = 0.91 to 1.61, *p* = 0.18, *I* ^2^ = 0%). However, at week 4, the cure rates of TGP combined with H1-antihistamine were significantly higher than those of H1-antihistamine alone (RR = 1.43, 95%CI = 1.26 to 1.61, *p* < 0.00001, *I* ^2^ = 0%). Moreover, the combination of TGP and H1-antihistamine was still superior to H1-antihistamine alone in improving the cure rate at week 8 (RR = 1.54; 95%CI = 1.31 to 1.80, *p* < 0.00001, *I* ^2^ = 39%) and week 12 (RR = 1.52, 95%CI = 1.28 to 1.80, *p* < 0.00001, *I* ^2^ = 0%) (Figure 5).

#### 3.4.2 Total efficacy rate

**FIGURE 6:**
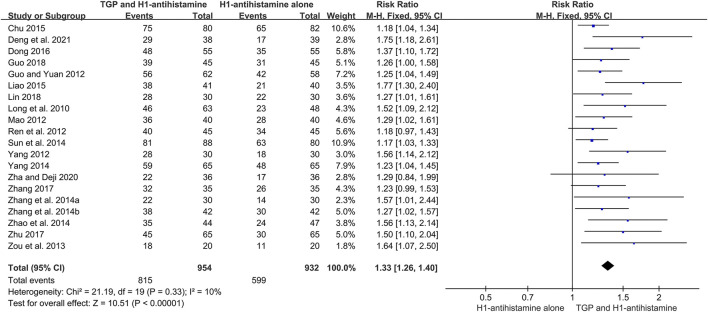
Forest plot of total efficacy rates between TGP combined with H1-antihistamine and H1-antihistamine alone.

There were 20 trials (Long et al., 2010; Guo and Yuan, 2012; Mao, 2012; Ren et al., 2012; Yang, 2012; Zou et al., 2013; Zhang et al., 2014a; Zhang et al., 2014b; Sun et al., 2014; Yang, 2014; Zhao et al., 2014; Chu, 2015; Liao, 2015; Dong, 2016; Zhang, 2017; Zhu, 2017; Guo, 2018; Lin, 2018; Zha and Deji, 2020; Deng et al., 2021) reporting total efficacy rate as an efficacy outcome, and 1886 patients were involved. Because of little heterogeneity (*I* ^2^ = 10%, *p* = 0.33), a fixed-effect model was performed. The pooled results displayed that in comparison with H1-antihistamine alone, TGP as an add-on treatment could significantly improve the total efficacy rate (RR = 1.33, 95%CI = 1.26 to 1.40, *p* < 0.00001) (Figure 6).

**FIGURE 7:**
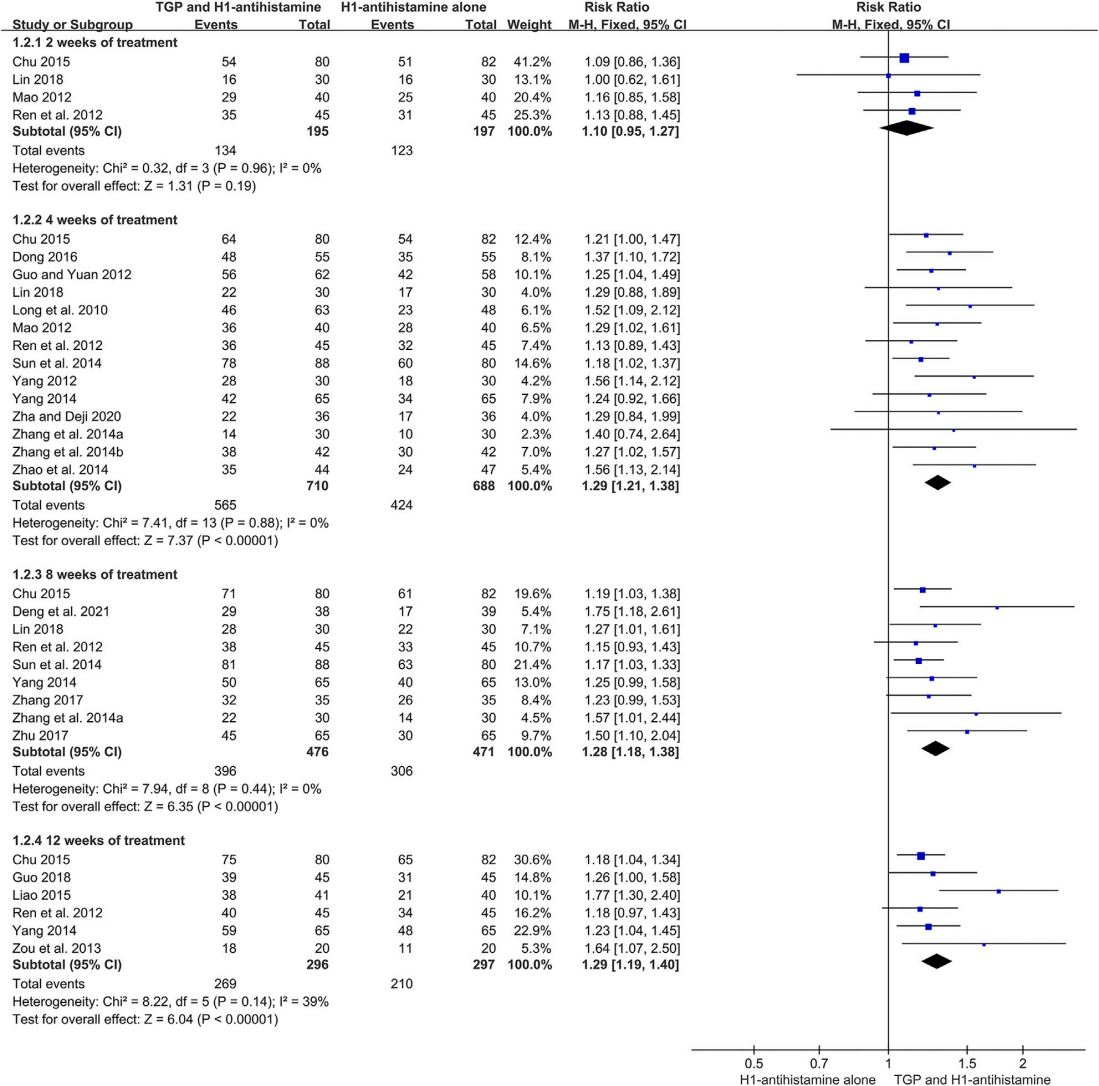
Forest plots of total efficacy rates at week 2, week 4, week 8, and week 12.

In addition, a subgroup analysis was also conducted based on different treatment durations. The results displayed that the total efficacy rates between two groups were not statistically different at week 2 (RR = 1.10, 95%CI = 0.95 to 1.27, *p* = 0.19, *I* ^2^ = 0%). However, at week 4, the total efficacy rates of the combination therapy were significantly higher than those of monotherapy (RR = 1.29, 95%CI = 1.21 to 1.38, *p* < 0.00001, *I* ^2^ = 0%). Furthermore, the improvement of the combination therapy was maintained at week 8 (RR = 1.28, 95%CI = 1.18 to 1.38, *p* < 0.00001, *I* ^2^ = 0%) and week 12 (RR = 1.29, 95%CI = 1.19 to 1.40, *p* < 0.00001, *I* ^2^ = 39%) (Figure 7).

### 3.5 Secondary outcomes

#### 3.5.1 UAS7

One trial (Liang et al., 2013) recruited 57 patients and used UAS7 to evaluate the disease severities between two groups during the treatment. The result showed that the scores between two groups were not significantly different at week 4 (MD = −0.59, 95%CI = −2.40 to 1.22, *p* = 0.52). However, the UAS7 score of TGP combined with ebastine was significantly lower than that of ebastine alone at week 8 (MD = −4.01, 95%CI = −6.43 to −1.59, *p* = 0.001) and week 12 (MD = −4.03, 95%CI = −6.62 to −1.44, *p* = 0.002). Moreover, after 4 weeks of follow-up, the combination therapy was still superior to monotherapy in reducing UAS7 score (MD = −8.69, 95%CI = −11.52 to −5.86, *p* < 0.00001) (Supplementary Figure S1).

#### 3.5.2 Recurrence rate

**FIGURE 8:**
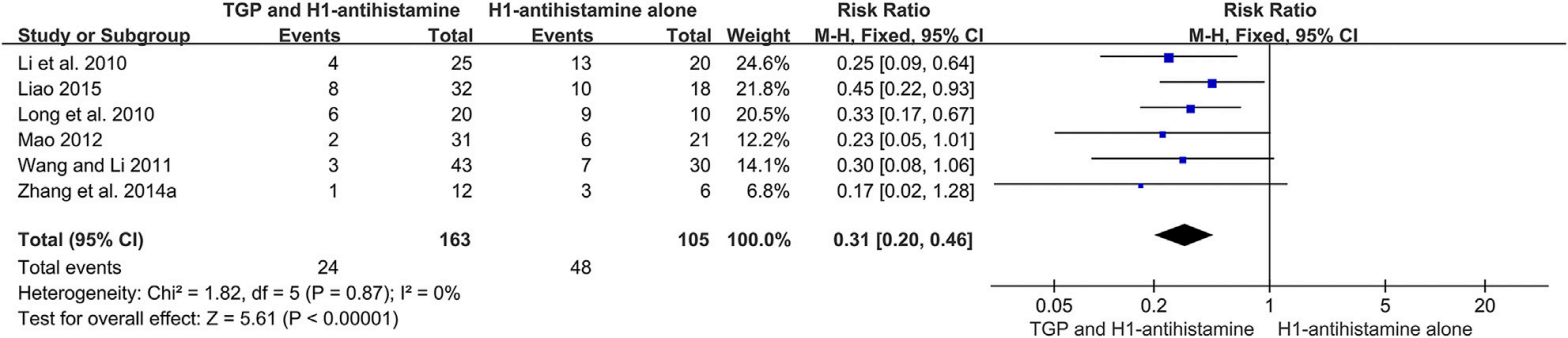
Forest plot of recurrence rates between TGP combined with H1-antihistamine and H1-antihistamine alone.

Six studies (Li et al., 2010; Long et al., 2010; Wang and Li, 2011; Mao, 2012; Zhang et al., 2014a; Liao, 2015) conducted a 1-month follow-up after drug withdrawal, and 268 patients were included. Due to no significant heterogeneity (*I* ^2^ = 0%, *p* = 0.87), a fixed-effect model was used. The pooled results revealed that TGP combined with H1-antihistamine could significantly decrease the recurrence rate in comparison with H1-antihistamine alone (RR = 0.31, 95%CI = 0.20 to 0.46, *p* < 0.00001) (Figure 8).

#### 3.5.3 The level of IgE in serum

**FIGURE 9:**
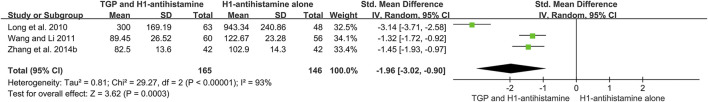
Forest plot of the levels of IgE in serum between TGP combined with H1-antihistamine and H1-antihistamine alone.

The levels of IgE in serum were measured in three studies (Long et al., 2010; Wang and Li, 2011; Zhang et al., 2014b), and 311 patients were involved. Due to different units of test methods among three studies, SMD was used. Because a significant heterogeneity (*I* ^2^ = 93%, *p* < 0.00001) was observed, which might be attributed to different test methods or baselines of disease severity, a random-effect model was performed. The pooled results showed that after 4 weeks of treatment, the levels of IgE in serum of TGP combined with H1-antihistamine were significantly lower than those of H1-antihistamine alone (SMD = −1.96, 95%CI = −3.02 to -0.90, *p* = 0.0003) (Figure 9).

#### 3.5.4 AEs

**FIGURE 10:**
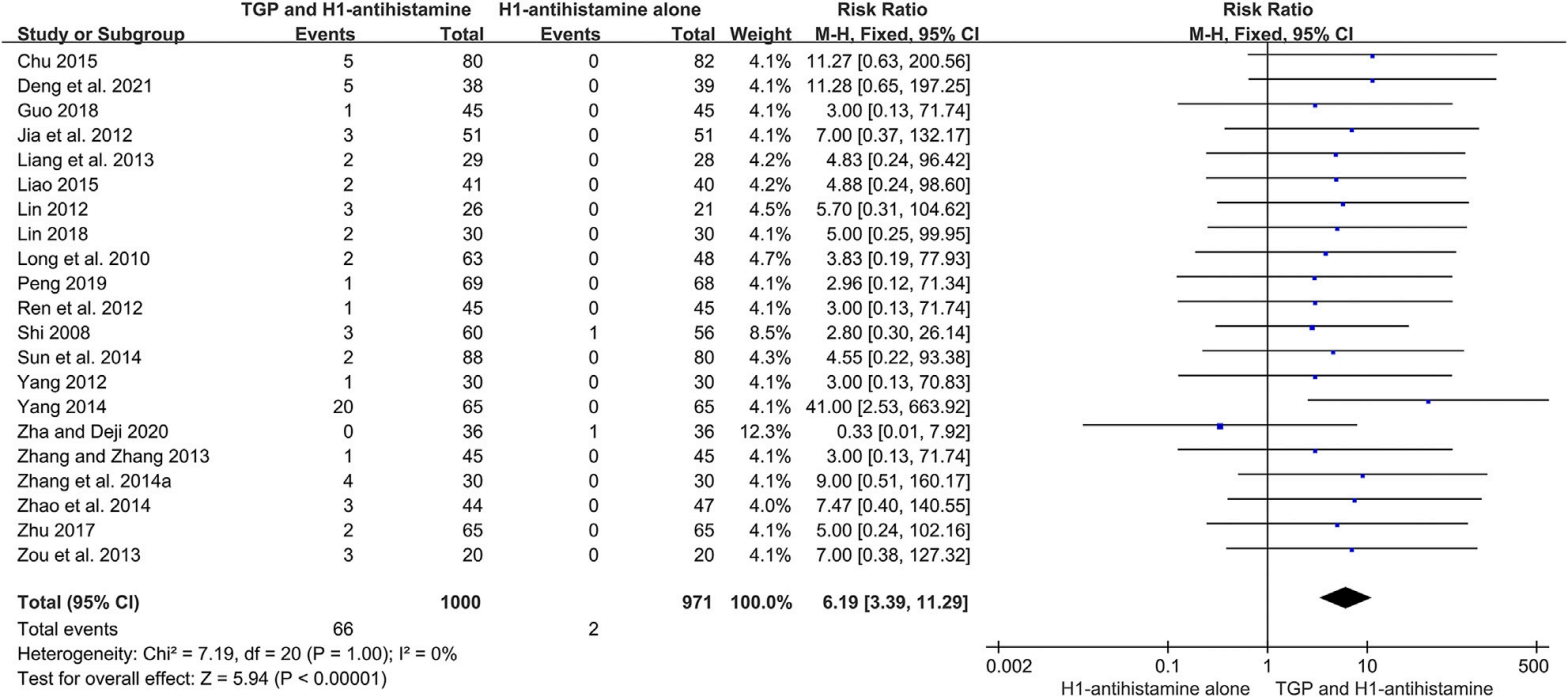
Forest plot of the incidences of diarrhea between TGP combined with H1-antihistamine and H1-antihistamine alone.

A total of 26 studies (Shi, 2008; Long et al., 2010; Guo and Yuan, 2012; Jia et al., 2012; Lin, 2012; Mao, 2012; Ren et al., 2012; Yang, 2012; Liang et al., 2013; Zhang and Zhang 2013; Zou et al., 2013; Zhang et al., 2014a; Sun et al., 2014; Xie, 2014; Yang, 2014; Zhao et al., 2014; Chu, 2015; Liao, 2015; Dong, 2016; Zhu, 2017; Guo, 2018; Lin, 2018; Peng, 2019; Cen, 2020; Zha and Deji, 2020; Deng et al., 2021) reported the detailed AEs during 4–12 weeks of treatment, and 2,631 patients were included. The incidences of AEs between two groups were low, and the symptoms of most AEs were mild and tolerable. The most common AE was diarrhea, which could relieve spontaneously or disappear after reducing the dosage of TGP. Due to no significant heterogeneity (*I* ^2^ = 0%, *p* = 1.00), a fixed-effect model was conducted. The pooled results showed that in comparison with H1-antihistamine alone, TGP as an add-on treatment significantly increased the incidence of diarrhea (RR = 6.19, 95%CI = 3.39 to 11.29, *p* < 0.00001) (Figure 10). Other common AEs included drowsiness, dry mouth, dizziness, and weakness. The pooled results showed that there were no significant differences between two groups in the incidences of drowsiness (RR = 0.73, 95%CI = 0.49 to 1.08, *p* = 0.12, *I* ^2^ = 0%), dry mouth (RR = 0.71, 95%CI = 0.38 to 1.35, *p* = 0.30, *I* ^2^ = 0%), dizziness (RR = 1.42, 95%CI = 0.63 to 3.20, *p* = 0.40, *I* ^2^ = 0%), and weakness (RR = 0.55, 95%CI = 0.19 to 1.56, *p* = 0.26, *I* ^2^ = 0%) (Supplementary Figure S2–5).

In terms of laboratory tests, 10 studies (Long et al., 2010; Lin, 2012; Mao, 2012; Yang, 2012; Liang et al., 2013; Zou et al., 2013; Sun et al., 2014; Xie, 2014; Dong, 2016; Deng et al., 2021) closely monitored blood routine, liver function, and renal function during the treatment, and the results of 900 patients in two groups were normal. Moreover, 840 patients in nine trials (Long et al., 2010; Lin, 2012; Mao, 2012; Liang et al., 2013; Zou et al., 2013; Sun et al., 2014; Xie, 2014; Dong, 2016; Deng et al., 2021) and 40 patients in one trial (Zou et al., 2013) conducted urine routine and stool routine tests, respectively, and no abnormal event was reported in two groups.

In addition, seven studies (Lin, 2012; Mao, 2012; Liang et al., 2013; Sun et al., 2014; Xie, 2014; Dong, 2016; Deng et al., 2021) conducted ECG before and after the treatment. A total of 376 patients in the test groups and 313 patients in the control groups were included. The results of ECG showed that there were no clinically significant changes in two groups.

### 3.6 Publication bias

**FIGURE 11:**
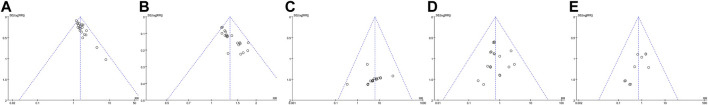
Funnel plots for publication bias. **(A)** cure rate; **(B)** total efficacy rate; **(C)** the incidence of diarrhea; **(D)** the incidence of drowsiness; **(E)** the incidence of dry mouth.

Publication biases of five outcomes were evaluated by funnel plot, Egger’s test and Begg’s test. In terms of cure rate, total efficacy rate, and the incidence of dry mouth, all funnel plots were significantly asymmetrical by visual assessment (Figure 11), suggesting a large possibility of publication bias. Furthermore, the results of Egger’s test (*p* < 0.001 for cure rate; *p* < 0.001 for total efficacy rate; *p* = 0.005 for the incidence of dry mouth) and Begg’s test (*p* = 0.001 for cure rate; *p* < 0.001 for total efficacy rate; *p* = 0.01 for the incidence of dry mouth) confirmed the significant publication biases for them. Instead, the nearly symmetrical funnel plot (Figure 11) and the negative results of Egger’s test (*p* = 0.795) and Begg’s test (*p* = 0.581) demonstrated that there was no obvious publication bias for the incidence of drowsiness. For the incidence of diarrhea, although the nearly symmetrical funnel plot (Figure 11) and the negative result of Egger’s test (*p* = 0.357) supported a small possibility of publication bias, the positive result of Begg’s test (*p* < 0.001) raised the risk of publication bias. The opposite results might be attributed to different powers between two tests under the condition of small sample studies and the limited number of included patients. Due to relatively low powers of two tests, the negative result could not absolutely exclude publication bias. It was still possible to have publication bias for the incidence of diarrhea. The Egger’s tests and Begg’s tests for five outcomes are shown in Supplementary Figure S6,7.

### 3.7 Sensitivity analysis

Sensitivity analysis was conducted to assess the stability of the results. After sequentially omitting each study at a time, there was no significant change in these results. Moreover, after changing the effect measure to OR, the pooled results of dichotomous data, including cure rate, total efficacy rate, recurrence rate, and the incidences of diarrhea, drowsiness, dry mouth, dizziness, and weakness, were not significantly different from those calculated by RR. Therefore, the results in this meta-analysis were robust.

### 3.8 GRADE assessment

The evidence qualities of ten outcomes were graded by using GRADE system, including cure rate, total efficacy rate, UAS7, recurrence rate, the levels of IgE in serum, and the incidences of diarrhea, drowsiness, dry mouth, dizziness, and weakness. Because of poor methodological quality, potential publication bias, and small number of participants, the qualities of evidence of them were evaluated to be “moderate” or “low”. The GRADE evidence profiles are shown in Table 2.

**TABLE 2:** GRADE summary of outcomes for TGP as an add-on treatment for CU in a adolescents and adults.

Outcomes	Number of studies	Number of patients		Effect		Quality of the evidence (GRADE)
		TGP + H1-antihistamine	H1-antihistamine	Relative (95%CI)	Absolute	
Cure rate	20	498/979 (50.9%)	314/953 (32.9%)	RR 1.54 (1.39–1.71)	178 more per 1,000 (from 128 more to 234 more)	⊕⊕○○ Low^a^ ^,^ ^b^
Total efficacy rate	20	815/954 (85.4%)	599/932 (64.3%)	RR 1.33 (1.26–1.40)	212 more per 1,000 (from 167 more to 257 more)	⊕⊕○○ Low^a^ ^,^ ^b^
UAS7	1	29	28	-	MD 4.03 lower (6.62–1.44 lower)	⊕⊕○○ Low^a^ ^,^ ^c^
Recurrence rate	6	24/163 (14.7%)	48/105 (45.7%)	RR 0.31 (0.20–0.46)	315 fewer per 1,000 (from 247 fewer to 366 fewer)	⊕⊕○○ Low^a^ ^,^ ^d^
The levels of IgE in serum	3	165	146	-	SMD 1.96 lower (3.02–0.90 lower)	⊕⊕○○ Low^a^ ^,^ ^c^
Incidence of diarrhea	21	66/1,000 (6.6%)	2/971 (0.21%)	RR 6.19 (3.39–11.29)	11 more per 1,000 (from 5 more to 21 more)	⊕⊕○○ Low^a^ ^,^ ^b^
Incidence of drowsiness	20	39/1,028 (3.8%)	53/1,011 (5.2%)	RR 0.73 (0.49–1.08)	14 fewer per 1,000 (from 27 fewer to 4 more)	⊕⊕⊕○ Moderate^a^
Incidence of dry mouth	11	13/640 (2.0%)	19/627 (3.0%)	RR 0.71 (0.38–1.35)	9 fewer per 1,000 (from 19 fewer to 11 more)	⊕⊕○○ Low^a^ ^,^ ^b^
Incidence of dizziness	5	12/290 (4.1%)	8/290 (2.8%)	RR 1.42 (0.63–3.20)	12 more per 1,000 (from 10 fewer to 61 more)	⊕⊕⊕○ Moderate^a^
Incidence of weakness	3	4/188 (2.1%)	8/177 (4.5%)	RR 0.55 (0.19–1.56)	20 fewer per 1,000 (from 37 fewer to 25 more)	⊕⊕⊕○ Moderate^a^

TGP, total glucosides of paeony; RR, risk ratio; MD, mean difference; SMD, standardized mean difference; CI, confidence interval.

a Poor methodological quality.

b Potential publication bias.

c The total number of patients less than 400.

d The total number of patients less than 300.

## 4 Discussion

Despite some advances in the treatment of CU in recent years, the balance among efficacy, safety and cost is a question that both clinicians and patients need to consider carefully. As an available and acceptable drug in China, TGP has been widely used for some inflammatory skin diseases, such as psoriasis, alopecia areata, and CU (Yang et al., 2012; Zheng et al., 2019). In this meta-analysis, the pooled results from 30 eligible studies showed that TGP could provide an effective and safe alternative treatment for adolescent and adult patients with CU.

Although the pathogenesis of CU has not been fully understood yet, the abnormal activation and degranulation of mast cells and basophils play an important role in the development of CU. In addition, eosinophils and T cells are also involved (He et al., 2021). TGP contains more than 15 constituents, including paeoniflorin, albiflorin, oxypaeoniflorin and benzoylpaeoniflorin, which could modulate the function and activation of immune cells. *In vitro* studies, paeoniflorin could decrease the phosphorylation levels of Lyn, PKC, Akt, P38, Erk1/2, and PLC in human mast cells and inhibit the release of histamine, 5-HT, IL-8, and TNF-α (Zhang et al., 2020). Moreover, it could also increase the expressions of FoxP3 and IL-10 and decrease the level of IL-17 in naive CD4^+^ T cells via dendritic cells (Zheng et al., 2020). *In vivo* studies, paeoniflorin could significantly alleviate scratching behavior, decrease the levels of IgE, leukotriene B4 and histamine in serum, and reduce the number of mast cells in the skin (Peng et al., 2022). Besides, it also inhibited the production and release of IL-23 and increased the autophagic activity via LKB1/AMPK-α pathway to improve urticarial lesions (Guo et al., 2021). Our meta-analysis showed that TGP as an add-on treatment could significantly decrease the level of IgE in serum in comparison with H1-antistamine alone. It was consistent with the results of vitro and vivo studies, and also indicated the anti-inflammatory and immunomodulatory effects of TGP for the treatment of CU.

With regard to efficacy, the pooled results revealed that TGP combined with H1-antihistamine was superior to H1-antihistamine alone in cure rate, total efficacy rate, and UAS7 score, demonstrating that TGP as an add-on treatment is effective for patients with CU. Furthermore, in terms of different treatment durations, there were no statistical differences on cure rates and total efficacy rates between two groups after 2 weeks of treatment, while the significant improvement in the combination therapy groups was observed at week 4, and the therapeutic advantage was maintained at week 8 and week 12. This phenomenon showed that TGP could not produce therapeutic effect immediately, and it needs to be continuously used for 4 weeks or more to achieve effect. On the other hand, UAS7 scores between two groups were not significantly different at week 4. However, at week 8 and week 12, the score of the combination therapy was significantly reduced in comparison with monotherapy. The discrepancy between cure rate or total efficacy rate and UAS7 score might be attributed to only one included trial and the small number of included patients, which decreased the precision of effect estimate. In fact, it still supported the slow action of TGP for the treatment of CU.

Meanwhile, after drug withdrawal and 1 month follow-up, the combination therapy was superior to monotherapy in recurrence rate and UAS7 score, which also reflected the effect of TGP on regulating immunity balance on the whole body. The above results showed that TGP as an add-on treatment could remarkably relapse disease recurrence and reduce disease aggravation. However, the included patients in these outcomes were limited, and more clinical trials focusing on the prognosis of CU after the treatment of TGP could be conducted in the future.

In terms of safety, the results of this meta-analysis showed that the incidence of diarrhea was significantly higher in the TGP plus H1-antihistamine groups, which was consistent with other studies (Zheng et al., 2019). The reason of diarrhea is that TGP could accelerate the peristalsis of gastrointestinal tract. However, the symptom for most patients is mild and tolerable, and does not cause organic damage to the gastrointestinal tract. Therefore, when diarrhea is encountered during the treatment of TGP, most patients could continue the treatment or reduce the dosage of TGP if necessary. In addition, the incidences of other common AEs, including drowsiness, dry mouth, dizziness, and weakness, were not significantly different between two groups. These symptoms might be considered as the side effects of H1-antihistamine. The above data showed that the safety of TGP was satisfied and acceptable.

On the other hand, blood and urine routines, liver and kidney functions, and ECG were closely monitored in some studies, and no significant clinical change was observed in two groups during the treatment. These results indicated that TGP was not characterized with obvious hematotoxicity, hepatotoxicity, nephrotoxicity and cardiotoxicity. In fact, TGP as adjuvant therapy is also applied to treat rheumatoid arthritis and diabetic kidney disease. Due to the anti-inflammatory and antioxidant effects, TGP could alleviate hepatic dysfunction, leukopenia and albuminuria (Zhu et al., 2016a; Liu et al., 2021). Therefore, TGP combined with H1-antistamine is a safe treatment for CU, and does not require to conduct regular laboratory tests during the treatment.

However, there are some limitations in this meta-analysis. Firstly, all included studies were conducted in China, and only Chinese patients were included, which might limit the external promotion of the evidence. Secondly, the suboptimal methodological quality of all included studies affected the reliability of results and the quality of evidence. Almost all studies did not describe the details on allocation concealment and blinding, and a few studies reported the methods of random sequence generation. Thirdly, some outcomes had small number of studies and participants, such as UAS7 score and recurrence rate, which might restrict the precision of evidence. Finally, the treatment durations of all included studies were not beyond 12 weeks, and there is no evidence on the efficacy and safety of long-term use of TGP for CU. Therefore, the pooled results of this meta-analysis should be interpreted cautiously, and high-quality and long-term RCTs are strongly needed in the future to confirm and update the evidence.

## 5 Conclusion

This meta-analysis demonstrated that TGP as an add-on treatment could provide a good effect for CU in adolescents and adults with mild and tolerable adverse events. However, more high-quality and long-term clinical trials are required in the future to provide more high-quality evidence for clinical practice.

## Data Availability

The original contributions presented in the study are included in the article/Supplementary Material, further inquiries can be directed to the corresponding author.
