# Supplementary material for: Efficacy and safety of total glucosides of paeony as an add-on treatment in adolescents and adults with chronic urticaria: A systematic review and meta-analysis
Source: Front Pharmacol. 2022 Sep 23;13:961371. doi: 10.3389/fphar.2022.961371 (PMC9574670; doi:10.3389/fphar.2022.961371)
Supplement: Supplementary file 1 [file DataSheet1.PDF]

	TGP + Ebastine			Ebastine alone				Mean Difference
Study or Subgroup	Mean	SD	Total	Mean	SD	Total	Weight	IV, Fixed, 95% CI
**1.3.1 4 weeks of treatment**								
Liang et al. 2013	14.17	3.44	29	14.76	3.52	28	100.0%	-0.59 [-2.40, 1.22]
**Subtotal (95% CI)**			**29**			**28**	**100.0%**	**-0.59 [-2.40, 1.22]**
Heterogeneity: Not applicable								
Test for overall effect: Z = 0.64 (P = 0.52)								
**1.3.2 8 weeks of treatment**								
Liang et al. 2013	9.28	4.59	29	13.29	4.72	28	100.0%	-4.01 [-6.43, -1.59]
**Subtotal (95% CI)**			**29**			**28**	**100.0%**	**-4.01 [-6.43, -1.59]**
Heterogeneity: Not applicable								
Test for overall effect: Z = 3.25 (P = 0.001)								
**1.3.3 12 weeks of treatment**								
Liang et al. 2013	5.83	4.44	29	9.86	5.46	28	100.0%	-4.03 [-6.62, -1.44]
**Subtotal (95% CI)**			**29**			**28**	**100.0%**	**-4.03 [-6.62, -1.44]**
Heterogeneity: Not applicable								
Test for overall effect: Z = 3.05 (P = 0.002)								
**1.3.4 4 weeks of follow-up after drug withdrawl**								
Liang et al. 2013	7.52	5.57	29	16.21	5.34	28	100.0%	-8.69 [-11.52, -5.86]
**Subtotal (95% CI)**			**29**			**28**	**100.0%**	**-8.69 [-11.52, -5.86]**
Heterogeneity: Not applicable								
Test for overall effect: Z = 6.01 (P < 0.00001)								

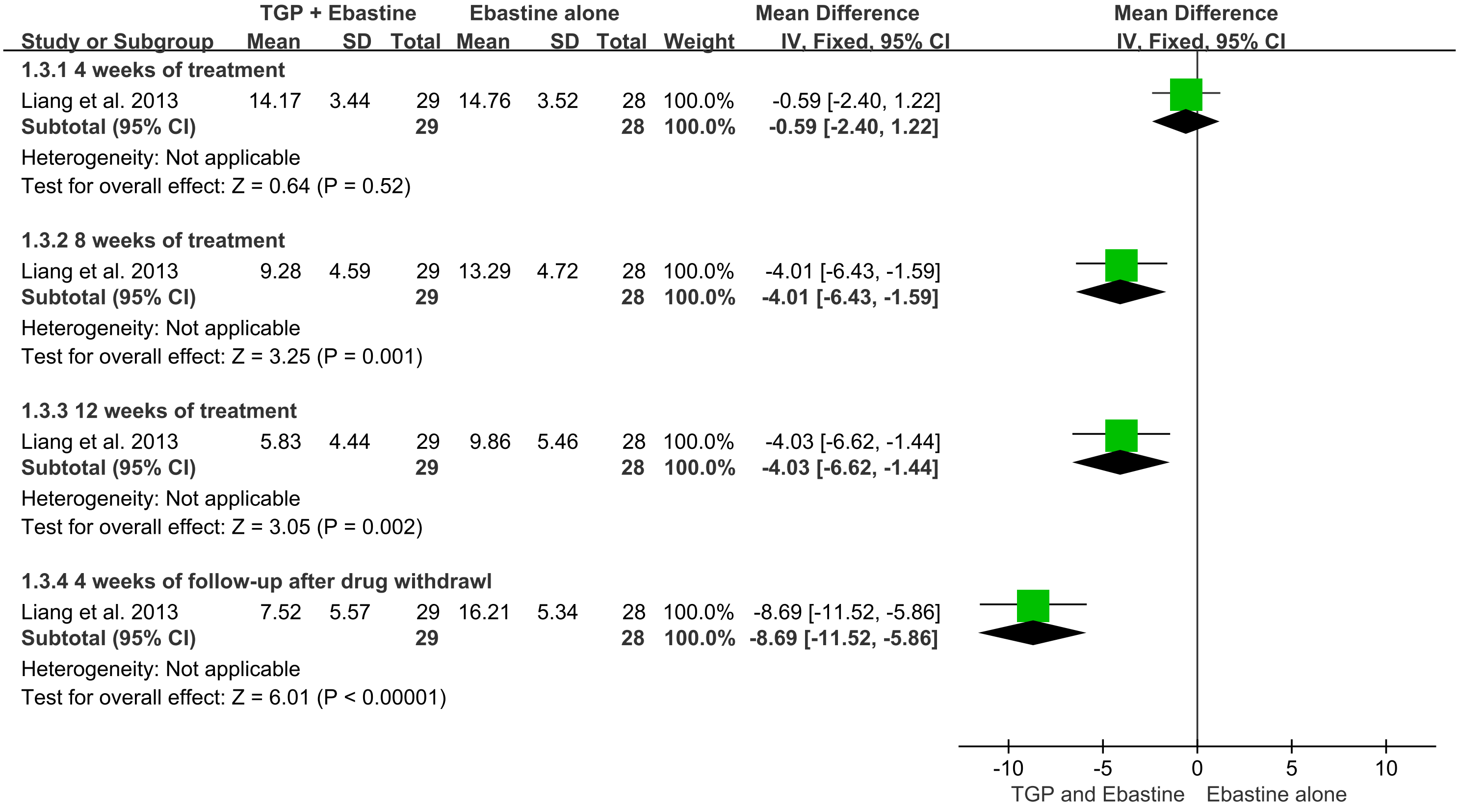

Supplementary Figure S1. Forest plots of UAS7 scores between TGP combined with ebastine and ebastine alone.

Study or Subgroup	TGP and H1-antihistamine Events	TGP and H1-antihistamine Total	H1-antihistamine alone Events	H1-antihistamine alone Total	Weight	Risk Ratio M-H, Fixed, 95% CI
Cen 2020	2	100	4	100	7.3%	0.50 [0.09, 2.67]
Chu 2015	1	80	1	82	1.8%	1.02 [0.07, 16.11]
Dong 2016	3	55	1	55	1.8%	3.00 [0.32, 27.96]
Guo 2018	0	45	2	45	4.6%	0.20 [0.01, 4.05]
Guo and Yuan 2012	5	62	2	58	3.8%	2.34 [0.47, 11.59]
Jia et al. 2012	2	51	3	51	5.5%	0.67 [0.12, 3.82]
Liao 2015	2	41	3	40	5.6%	0.65 [0.11, 3.69]
Lin 2012	1	26	2	21	4.1%	0.40 [0.04, 4.15]
Mao 2012	4	40	6	40	11.0%	0.67 [0.20, 2.18]
Ren et al. 2012	1	45	2	45	3.7%	0.50 [0.05, 5.32]
Shi 2008	4	60	6	56	11.4%	0.62 [0.19, 2.09]
Sun et al. 2014	0	88	1	80	2.9%	0.30 [0.01, 7.34]
Yang 2012	1	30	2	30	3.7%	0.50 [0.05, 5.22]
Yang 2014	4	65	6	65	11.0%	0.67 [0.20, 2.25]
Zha and Deji 2020	1	36	1	36	1.8%	1.00 [0.07, 15.38]
Zhang and Zhang 2013	2	45	1	45	1.8%	2.00 [0.19, 21.28]
Zhang et al. 2014a	1	30	2	30	3.7%	0.50 [0.05, 5.22]
Zhao et al. 2014	2	44	4	47	7.1%	0.53 [0.10, 2.77]
Zhu 2017	1	65	2	65	3.7%	0.50 [0.05, 5.38]
Zou et al. 2013	2	20	2	20	3.7%	1.00 [0.16, 6.42]
**Total (95% CI)**		**1028**		**1011**	**100.0%**	**0.73 [0.49, 1.08]**
Total events	39		53			

Heterogeneity: Chi² = 6.61, df = 19 (P = 1.00); I² = 0%

Test for overall effect: Z = 1.57 (P = 0.12)

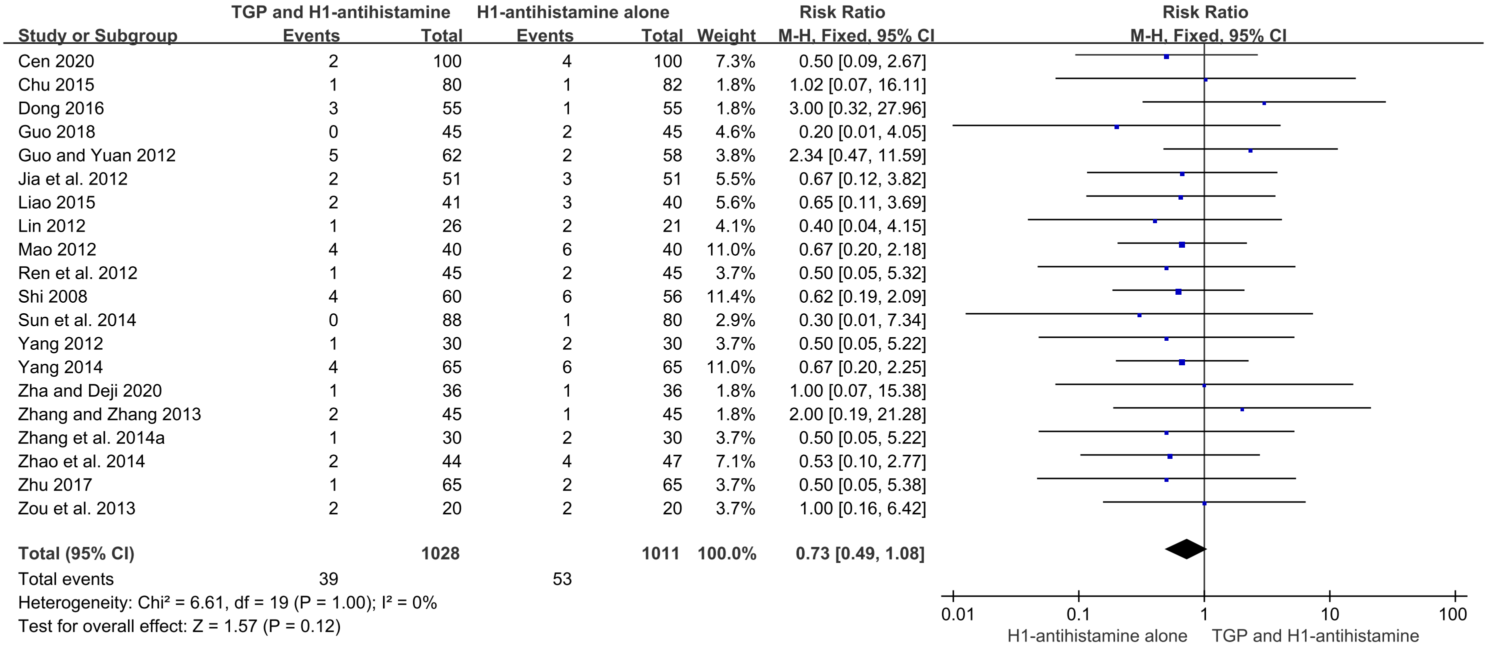

Supplementary Figure S2. Forest plot of the incidences of drowsiness between TGP combined with H1-antihistamine and H1-antihistamine alone.

	TGP and H1-antihistamine		H1-antihistamine alone			Risk Ratio
**Study or Subgroup**	**Events**	**Total**	**Events**	**Total**	**Weight**	**M-H, Fixed, 95% CI**
Cen 2020	2	100	3	100	13.9%	0.67 [0.11, 3.90]
Dong 2016	3	55	2	55	9.2%	1.50 [0.26, 8.63]
Guo 2018	0	45	2	45	11.5%	0.20 [0.01, 4.05]
Guo and Yuan 2012	0	62	2	58	11.9%	0.19 [0.01, 3.82]
Peng 2019	2	69	1	68	4.7%	1.97 [0.18, 21.23]
Ren et al. 2012	0	45	1	45	6.9%	0.33 [0.01, 7.97]
Sun et al. 2014	0	88	1	80	7.3%	0.30 [0.01, 7.34]
Yang 2012	1	30	2	30	9.2%	0.50 [0.05, 5.22]
Zha and Deji 2020	2	36	2	36	9.2%	1.00 [0.15, 6.72]
Zhang and Zhang 2013	0	45	1	45	6.9%	0.33 [0.01, 7.97]
Zhu 2017	3	65	2	65	9.2%	1.50 [0.26, 8.68]
**Total (95% CI)**		**640**		**627**	**100.0%**	**0.71 [0.38, 1.35]**
Total events	13		19			

Heterogeneity: Chi² = 4.46, df = 10 (P = 0.92); I^2 = 0%
Test for overall effect: Z = 1.03 (P = 0.30)

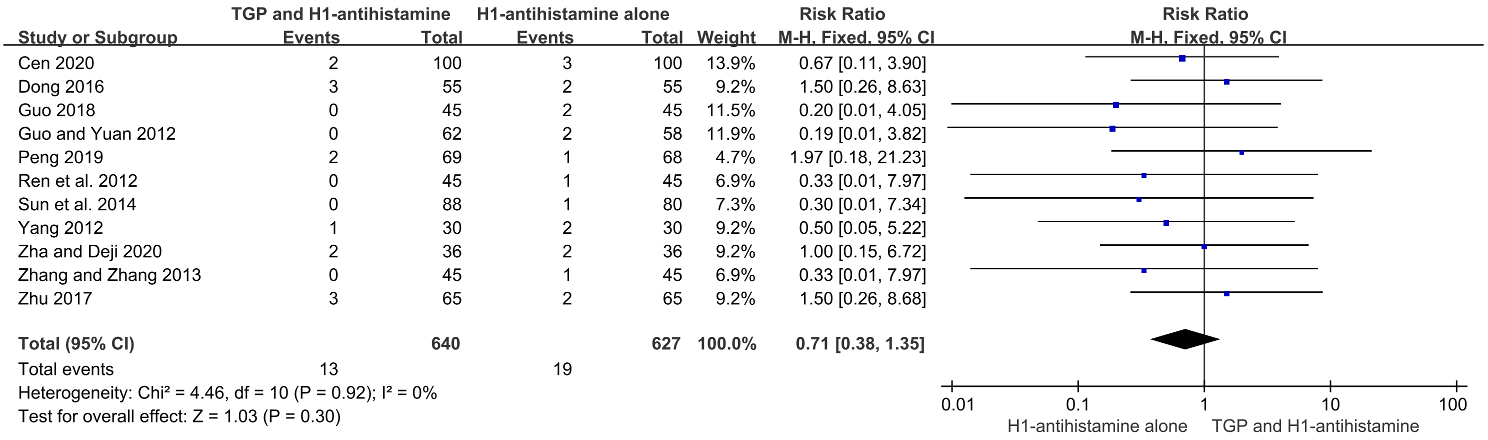

Supplementary Figure S3. Forest plot of the incidences of dry mouth between TGP combined with H1-antihistamine and H1-antihistamine alone.

	TGP and H1-antihistamine		H1-antihistamine alone			Risk Ratio
Study or Subgroup	Events	Total	Events	Total	Weight	M-H, Fixed, 95% CI
Cen 2020	3	100	4	100	42.1%	0.75 [0.17, 3.27]
Dong 2016	4	55	4	55	42.1%	1.00 [0.26, 3.80]
Guo 2018	2	45	0	45	5.3%	5.00 [0.25, 101.31]
Ren et al. 2012	1	45	0	45	5.3%	3.00 [0.13, 71.74]
Zhang and Zhang 2013	2	45	0	45	5.3%	5.00 [0.25, 101.31]
**Total (95% CI)**		**290**		**290**	**100.0%**	**1.42 [0.63, 3.20]**
Total events	12		8			

Heterogeneity: Chi² = 2.55, df = 4 (P = 0.64); I² = 0%

Test for overall effect: Z = 0.85 (P = 0.40)

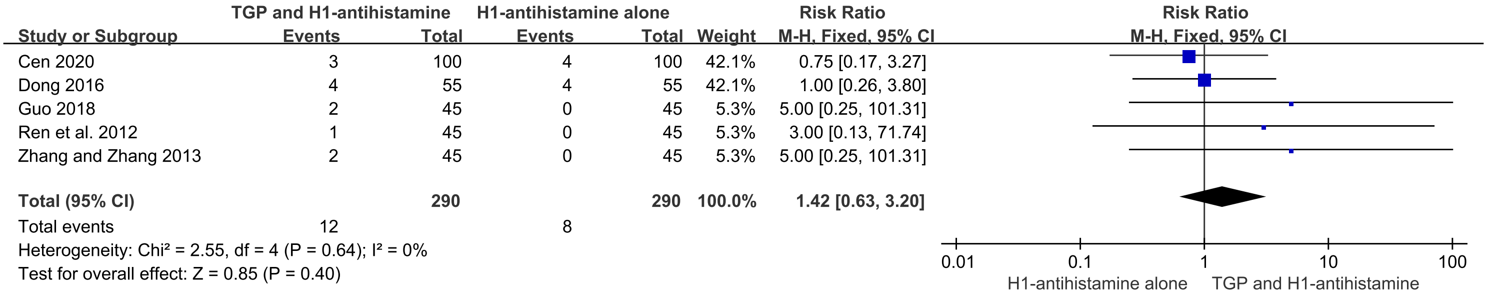

Supplementary Figure S4. Forest plot of the incidences of dizziness between TGP combined with H1-antihistamine and H1-antihistamine alone.

Study or Subgroup	TGP and H1-antihistamine Events	Total	H1-antihistamine alone Events	Total	Weight	Risk Ratio M-H, Fixed, 95% CI
Deng et al. 2021	4	38	5	39	54.3%	0.82 [0.24, 2.83]
Guo and Yuan 2012	0	62	2	58	28.4%	0.19 [0.01, 3.82]
Sun et al. 2014	0	88	1	80	17.3%	0.30 [0.01, 7.34]
**Total (95% CI)**		**188**		**177**	**100.0%**	**0.55 [0.19, 1.56]**
Total events	4		8			

Heterogeneity: Chi² = 1.03, df = 2 (P = 0.60); I^2 = 0%
Test for overall effect: Z = 1.12 (P = 0.26)

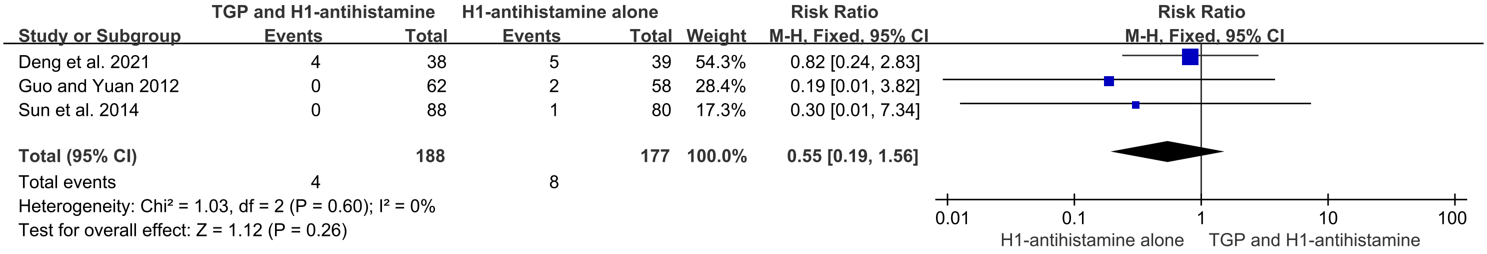

Supplementary Figure S5. Forest plot of the incidences of weakness between TGP combined with H1-antihistamine and H1-antihistamine alone.

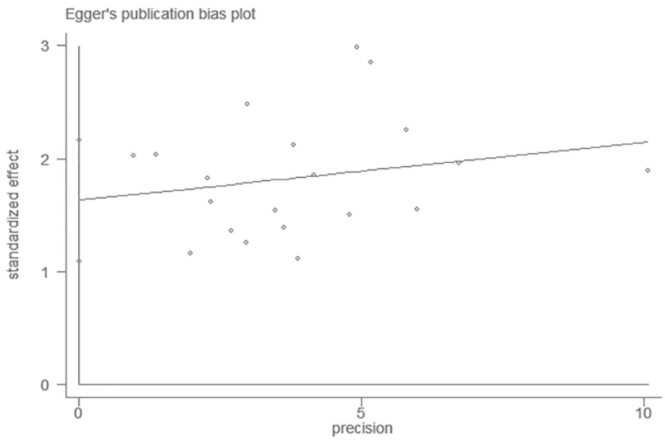

(A)

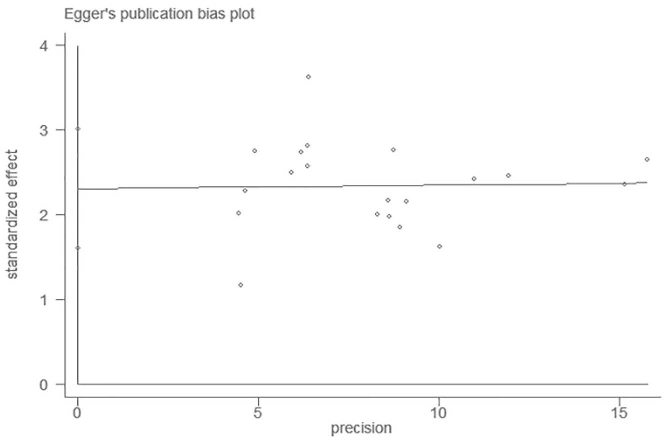

(B)

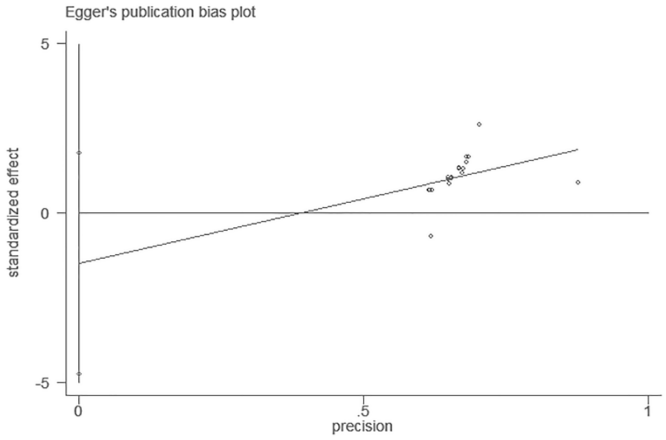

(C)

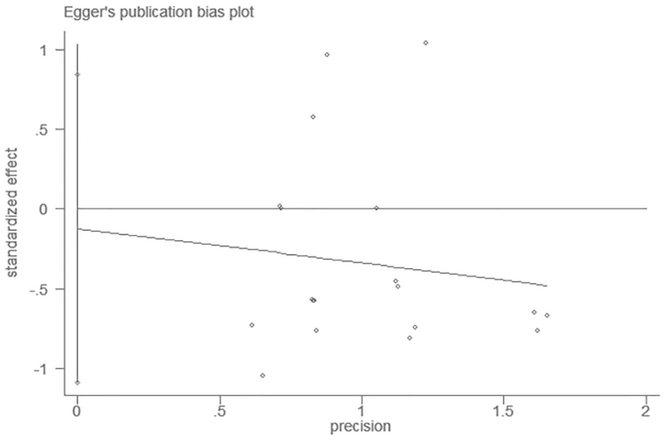

(D)

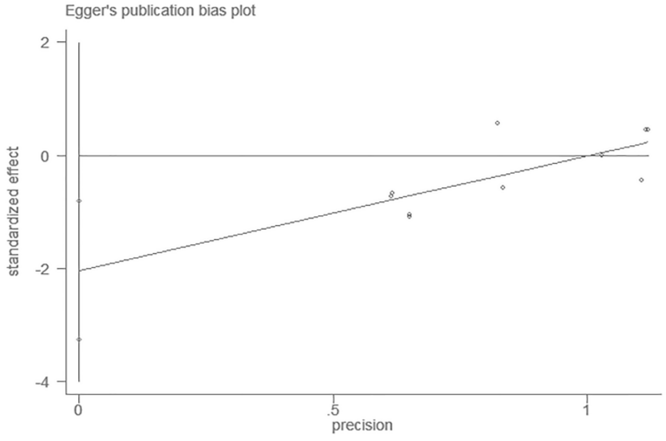

(E)

Supplementary Figure S6. Egger’s tests for publication bias. (A) cure rate; (B) total efficacy rate; (C) the incidence of diarrhea; (D) the incidence of drowsiness; (E) the incidence of dry mouth.

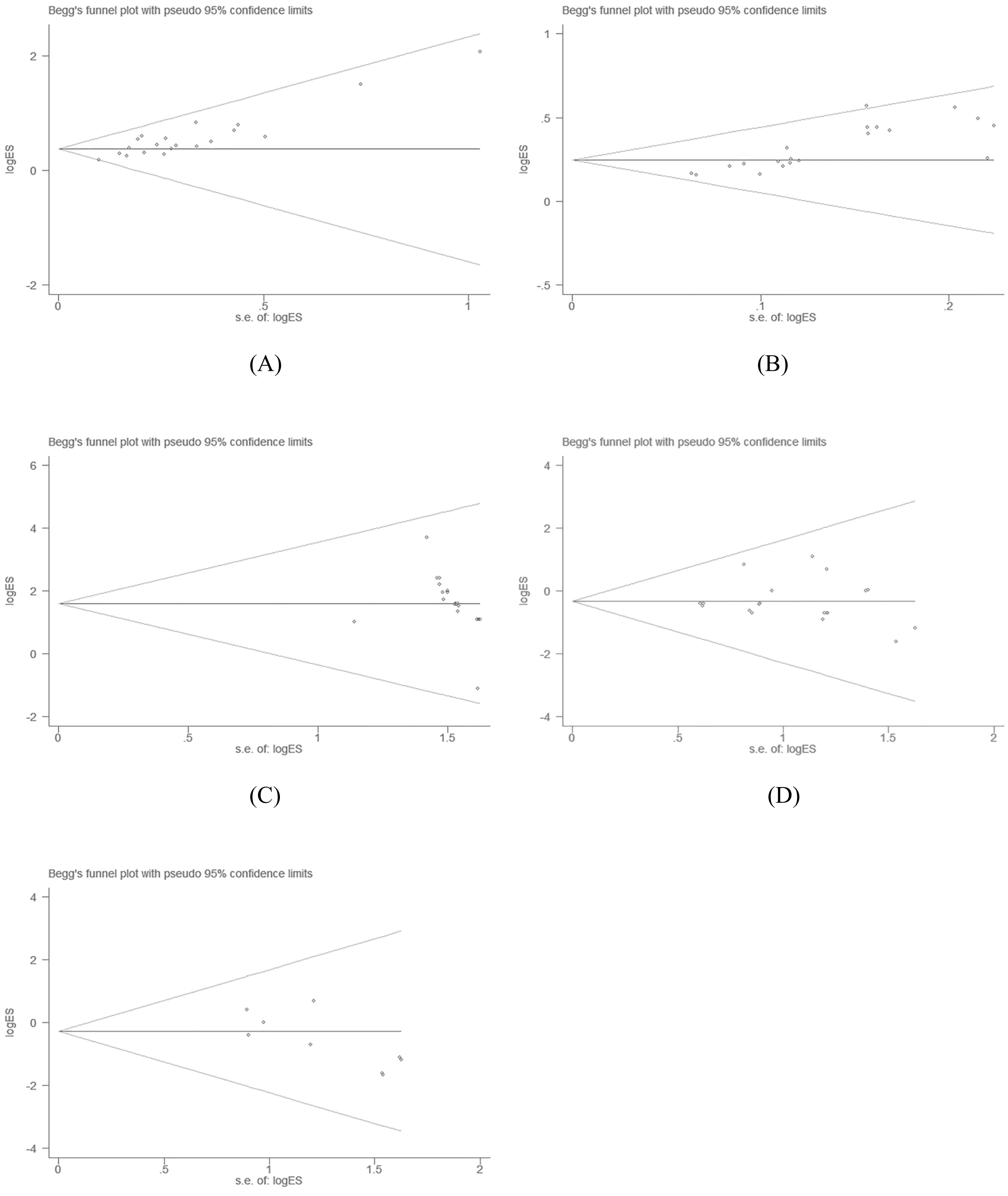

(E)

Supplementary Figure S7. Begg's tests for publication bias. (A) cure rate; (B) total efficacy rate; (C) the incidence of diarrhea; (D) the incidence of drowsiness; (E) the incidence of dry mouth.
